# Greater involvement of people living with HIV in health care

**DOI:** 10.1186/1758-2652-12-4

**Published:** 2009-03-14

**Authors:** Odetoyinbo Morolake, David Stephens, Alice Welbourn

**Affiliations:** 1 Positive Action for Treatment Access, Lagos, Nigeria; 2 Nossal Institute for Global Health, University of Melbourne, Victoria, Australia; 3 International Community of Women Living with HIV/AIDS, London, UK

## Abstract

Greater Involvement of People Living with HIV/AIDS represents a mobilising and an organising principle for the involvement of people living with HIV in program and policy responses. People with HIV have been at the forefront of designing and implementing effective HIV treatment, care and prevention activities. However, governments and health systems have yet to act to fully harness the potential and resources of people living with HIV in addressing the epidemic.

The lives and experiences of people living with HIV highlight the need for a shift in the existing paradigm of disease management. The high prevalence of HIV amongst health care providers in many countries, exacerbated by stigma towards those with HIV in the health care professions, is seriously undermining the capacity of health systems and signals the need to change the current nature of health care delivery. Moreover, the negative experiences of many people with HIV in relation to their health care as well as in their daily social interactions, coupled with the ever-limited current investment in treatment, care and support, demonstrate that the current system is drastically failing the majority of people with HIV. Current health management systems urgently need to be more effectively maximised, to increase the quality of standards of health care systems and services in resource poor countries. An integrated approach to health care based on a human rights framework, grounded in community realities and delivered in partnership and solidarity with people living with HIV, offers the most viable approach to overcoming the crisis of HIV in the health care system.

## Background

The year 2008 marked the 30^th ^anniversary of the Declaration of Alma Ata, which was a first attempt to articulate the goal of "public health for all" within a single policy framework [1]. This year also marked the 60^th ^anniversary of the Universal Declaration of Human Rights, as well as the establishment of the World Health Organization (WHO). These anniversaries remind us that access to health care and human rights are fundamental to our struggle to improve the health and well-being of people living with HIV; and that the attainment of the highest possible level of health is the most important world-wide social goal requiring the action and leadership of many sectors – social, economic and legal – in addition to the health sector. Indeed, all the Millennium Development Goals (MDGs) relate in one form or another to health. Unless the quality and breadth of health care systems around the world are systematically improved, we will not reach the targets set by the MDGs.

A lesser known, but equally important commemoration in 2008 was the 14^th ^anniversary of the principle of the Greater Involvement of People Living with HIV and AIDS (GIPA) in the response to the pandemic, [2]. It is deeply shameful that we still have so far to go in realizing the vision embodied in these declarations.

The authors of this paper also mark our own personal anniversaries of survival with HIV, and we acknowledge and salute the millions of people living with HIV around the world who continue to inspire us and who are the true authors of this story. We invite readers to come on a journey of what it means to be living with HIV and how it feels when our health systems let us down. We invite you to experience with us how it feels to know that as individuals we have so much to offer our countries, our health systems and our communities; how it feels for that offer to be ignored, forgotten or rejected; and how it feels to be stigmatised and criminalised as 'carriers of HIV', or treated as vectors of transmission. In this article we examine the crisis of HIV and its impact on health systems in resource poor settings. We also explore the glimmers of hope; the work that people with HIV around the world are doing to help reverse this crisis; and we offer an agenda to support and strengthen these efforts.

### The foundation of the Greater Involvement of People living with HIV (GIPA)

We begin with a glance back at the history of the self-empowerment and self-help movement of people with HIV. The foundational right of all people to participate in social, cultural and scientific activities is enshrined in the 1948 Universal Declaration of Human Rights, which states that "Everyone has the right freely to participate in the cultural life of the community, to enjoy the arts and to share in scientific advancement and its benefits." (Article 27, paragraph 1) [3]. The right to participate in health care systems and policies is also an important aspect of the normative content of Article 12 (the right to health) of the International Covenant on Economic, Social and Cultural Rights. The United Nations Committee on Economic Social and Cultural Rights has interpreted the right to health to include the participation of the population in all health-related decision-making at the community, national and international levels [4].

On May 2, 1983, the first candlelight march led and organised by people with HIV was held in San Francisco. The goal was to draw attention to the plight of those with HIV and AIDS, and remember those who had died. The march was led by people with HIV holding a banner with the slogan "Fighting for Our Lives", which became the motto of the People with AIDS Self-Empowerment Movement, and led to the drafting of the Denver Principles [5]. The Denver Principles are simple, but their significance has been profound. They articulate the key challenges in the lives of those living with HIV and the role of people with HIV themselves in overcoming such challenges by refusing to be victims and demanding to be involved.

The essential tenets of the Denver Principles are still relevant today. The right of people with HIV to participate as active and equal partners in the response to HIV and AIDS finds its most recent articulation in the GIPA principle. GIPA recognizes that the contribution of people with HIV at all levels and in all sectors is critical to ethical and effective national responses to the epidemic [6]. GIPA is important because it acknowledges the past contributions people with HIV have made and it provides a vision for the future in which people with HIV take their places as equal partners with governments, donors, health workers and others, working to stem the tide of the pandemic. GIPA also represents an organising principle from which we can shape our involvement as networks and groups connected at the national and international levels. GIPA serves as a necessary reminder of the importance of taking active control of our lives and our health.

The nobel laureate Professor Wole Soyinka wrote:*"The man dies in all who keep silent in the face of tyranny"*[7]. Everyday around the world, people with HIV continue to be inspired by this ideal. These individuals speak out on behalf of others, often at great risk, knowing that there are many people, especially women, who want to speak out about HIV, but cannot risk doing so for fear of risking their livelihoods and their children.

### The Role of people living with HIV in Health care systems

In its 2007 Framework for Action, WHO defines health systems as all organizations, people and actions whose primary interest is to promote, restore or maintain health [8]. Health systems include a mother caring for a sick child at home, private providers, behaviour change programmes, health insurance organizations, and occupational health and safety legislation. Health systems should provide health and health equity in ways that are responsive, financially fair and make the most efficient use of available resources. Health systems are a collective responsibility and, as the WHO Framework for Action title indicates, "Everybody's Business". We have learnt the hard way the truth of this statement, and that our health outcomes rely on a broad set of institutions and systems, as well as our communities and ourselves.

None of us living with HIV expected to have to learn so much so fast about things we never imagined we would need to face in life. Many of us – and many of our children who have seen the effects of HIV in their own lives – have either trained formally in the caring professions, including health, or have informally become so-called "patient experts" on all kinds of aspects of HIV prevention, treatment and care. "*Im*patient experts" would be a better way to describe us. We are perhaps the most health and treatment literate client body in the history of disease management and this fact alone is of fundamental importance to our role in HIV health care and treatment systems.

*"The key to successful treatment education and advocacy is remembering our own dignity as human beings and taking hold of the power of that truth. Taking care of our feelings is as important as understanding and treatment of the physical manifestations of this disease. We are our own cure..." Paisan Suwannawong *[9].

To understand the importance of the impact people with HIV have made and are yet to make in the response to HIV, we must acknowledge the place of people with HIV in developing and striving for the foundational and most effective responses to the epidemic. These include: the concept of safe sex; the pioneering of harm reduction; the value of peer education; progressive and inclusive policy and law; the undocumented burden of caring; the insight into treatment; the tireless work on prevention education; the creation of supportive organizations and groups, often in extremely hostile environments; and the human rights campaigning and sacrifices of many people living with HIV and AIDS.

People with HIV also have been instrumental in reshaping critical areas of HIV research. From basic science to behavioural and social research, the experience and knowledge of people with HIV has been essential to setting a direction that is relevant, and that respects the rights of research participants and can be translated into interventions which make a practical and immediate difference.

Treatment activism has helped to change the way in which drug trials are conducted and the approval process for the release and access to new drugs [10,11]. Our engagement has also led to a more open environment and the democratization of scientific knowledge. We have pushed the scientific and publishing industry to create new pathways for the sharing of knowledge, and we have created our own. In social research our involvement helps to build the trust between researchers and highly stigmatized and marginalized communities, helping researchers learn from the experience and insight of communities.

### HIV and the health system crisis

HIV is decimating the health workforce in many countries. The statistics are chilling, as shown by reports emerging from South Africa and Uganda [12,13]. At a recently concluded workshop for bank staff conducted in Nigeria by Positive Action for Treatment Access Nigeria, 70% of participants believed that health care providers with HIV should be prevented from conducting active clinical work and invasive procedures [14]. Problems also exist in so-called "developed" countries. In the United Kingdom, many staff living with HIV employed by the National Health Service are still terrified of their colleagues and managers finding out their HIV status [15]. In Europe, Young Positives Co-Founder Raoul Fransen recalled the appalling way he was treated in medical school when he disclosed his positive status. Things got so bad he decided to take his considerable talents into public health instead – luckily for all of us [16]. In Africa, our dear sister and friend, Yinka Jegede, chose to stay and fight for her right to remain in school and become a nurse when she was diagnosed while still a 19 year old nursing student [17].

Official recruitment of HIV-positive people into the health system is limited, and where it does happen, HIV-positive people are mainly employed as peer supporters and counsellors. These jobs are important, but the lack of a proactive approach to recruiting and supporting staff in more senior positions reflects and reinforces entrenched stigma and discrimination. WHO is at last beginning to challenge the paucity of opportunities for people with HIV through its "Treat, Train and Retain" initiative, echoing the important lead of UNESCO/EI-EFAIDS in supporting teachers living with HIV [18]. The GIPA statement requires investment in people with HIV in order to establish the conditions in which we can become meaningfully involved. Health workers living with HIV must be affirmed and encouraged. Opportunities must be created to help them become the very best they can be.

Until very recently, doubts about the capacities of health systems and people with HIV to manage anti-retroviral drugs, particularly in Africa, delayed the introduction of treatment and led to the death of many. These doubts were at best ill conceived and more often a thinly veiled racist commentary on African people. Such attitudes should not be allowed to block efforts to provide the best quality care and the latest technology, including pap smears, microbicides, and both female and male condoms. Yet people with HIV continue to suffer from drug stock-outs, and limited paediatric formula for their children. In 2008, three of the ten million people with HIV in need of antiretrovirals (ARVs) are actually able to access them [19].

For some, the risks of accessing HIV services may still outweigh the benefits. Entrenched judgements about sex workers, men who have sex with men, transgender people, drug users and women with HIV who choose to have babies – despite the minimal risk to their children, if proper health care is given – mean that quality care is often simply not available. If this is hard to believe, ask the women who have been coerced into having an abortion, or who have been sterilised. Ask the drug users who have been locked up and made to suffer forced detoxification and the loss of their liberty. Ask the men who have sex with men and transgender people who are routinely beaten and denied access to basic services [20,21].

### Vertical versus integrated health care for people with HIV

Health systems are still struggling to integrate HIV care with other services essential to supporting people living with HIV. TB, malaria, sexually transmitted infections, sexual and reproductive health, and mental health services need to be integrated with HIV services. HIV highlights how ineffective a vertical approach to any health issue is, as it seeks to squeeze people into boxes created by management systems and delineated by professional boundaries. This needs to be addressed urgently and immediately, as the lack of integration or unavailability of other essential services for people living with HIV cause yet more hardship. The overriding imperative of HIV prevention means, for example, that prevention of mother-to-child transmission programmes are designed not as comprehensive maternal and child health programmes, but with the primary aim of producing HIV-negative babies. They reconstruct women as biological vectors of disease transmission, rather than as individual sentient people with sexual and reproductive rights and human desires to be loving and caring mothers [22].

### Health systems, violence and criminalisation of HIV transmission

Amnesty International, Physicians for Human Rights and ICW have all documented the partner violence experienced by countless women after testing positive [23-25]. This has its effect on the physical, sexual and mental health of women, their children and babies. Women are now also subject to criminal sanctions for transmitting HIV (including to their children). We note that these legal developments can be understood as a partial extension of public health policies moving increasingly towards mandatory testing [26].

Psychosocial support for HIV-positive people is desperately needed around the world. A recent study in countries in Asia and the Pacific found that 36% of patients had evidence of depression [27]. Many abstracts published at the XVII International AIDS Conference in Mexico also document the widespread chronic depression experienced by children, women and men alike, who are either HIV-positive or living with a family member who has HIV [28]. Even well intended health interventions can undermine the broader health of people with HIV. A poster presentation at the same conference highlights the loss of autonomy and independence people experience as a consequence of participating in DOT/ART programs, thereby undermining adherence and regular clinic attendance [29].

In so many affected regions, and particularly in Africa, AIDS responses have been built around home based care established and developed by communities. As mothers, daughters, sisters and grandmothers, women carry the burden of care whatever the nature of the epidemic and are unheard, invisible, unpaid, and desperately overstressed. Girls too are deeply affected. They are the first to be pulled out of school to help their mothers cope, the first to have to use their bodies sexually to find alternative sources of income, and the first to be sent off to work in low paid jobs as house-servants, where they are frequently exploited, or married off to relieve the household of one less mouth to feed. A mapping exercise conducted by ICW and funded by WHO found that women living with HIV experience many types of social and economic barriers to accessing treatment, and to ensuring that they are able to adhere to treatment [30]. WHO has to date not reported these findings in its literature; preferring to publish reports simply stating that more women than men are accessing ARVs from public health centres [31].

### The work and achievements of people living with HIV

There are, however, many glimmers of hope and signs of change. Stepping Stones ("Paso a Paso" in Spanish) is a prevention initiative created out of personal positive experiences, founded on the basis of care, respect, support for and inclusion of everyone with HIV in a community [32]. The training package promotes gender equity, inter-generational respect and solidarity with HIV-positive people, within a human rights framework. Reports of reductions in gender violence using this programme are widespread from many different countries [33].

Many thousands of people with HIV are actively involved in prevention work and are not interested in spreading this virus. The international community responding to HIV should celebrate the work and sing the praises of the many people with HIV around the world who are doing their own bit, large or small, openly or undisclosed, to make the world a better place for all around them [34,35]. There are many examples, including the work of Dr Lydia Mungherera and her colleagues at the Mama's Club in Uganda, who were the proud recipients of the 2008 Red Ribbon Award for their great work in providing psycho-social support for young positive mothers in Uganda. Another example of positive leadership is Dr Jorge Saavedra Lopez, the openly positive director of Mexico's National AIDS Control Centre. These inspirational people have given us all so much. But these initiatives and courageous people, as well as others like them, are struggling to keep going. We need to rethink the response to make these islands of hope the mainstream, the norm and the expected.

### The way forward

We hope we have by now made the point that things will not change unless people with HIV are centrally involved. So we all need to step outside the predetermined management- and system-focused boxes, to develop proper *people-centred *not system-centred thinking and to create holistic projects which reflect real lives. This means understanding that a functioning health system is everybody's business. It means listening to, working with and supporting the meaningful involvement of people living with HIV to understand how issues like law, food or violence interconnect, and how they affect HIV, health and health systems. The work of the Salvation Army in developing human capacity development approaches, and of the Cambodian HIV/AIDS Education and Care Initiative, both of which support and build from community responses, provide a principled and grounded approach to HIV interventions which recognise that community expertise and experience is critical to success [36,37].

In addition to contributing critical work in our communities, people with HIV have begun to participate in the governance structures of the Global Fund and other international bodies, as well as in senior management and decision making roles. But huge gaps remain at the national level. Governments, donors, National AIDS Control Programmes, lawmakers and nongovernmental organization-led HIV programmes need to open their doors to us.

The WHO HIV Department is beginning to commit to a meaningful civil society consultation process, to recognise the non-biomedical dimensions of this pandemic, and respond to the health systems in a true holistic sense. We urge WHO to encourage states throughout the world to maintain data disaggregated by sex and age throughout health care systems, and to use the global leverage and leadership of WHO and the United Nations family to support people with HIV and challenge model laws that criminalise transmission of HIV [38,39]. We also desperately need Wellness Centres like those run by the Swaziland Nurses Association, which provide treatment, care support and counselling for health workers and their families [40,41]. These services are essential to keeping our highly valued health workers alive.

We need to ensure universal access to halt and eventually reverse the devastating losses to AIDS among all people living with HIV. This means Greater "*Investment*" in the "*Lives*" of People Living with – and directly affected by – HIV and AIDS. All partners need to recognise and adequately reward the roles people with HIV play and see us as far more than volunteers and recipients of services. This means enhancing our skills, and consulting with us when programmes and policies that affect our lives are being designed.

Funding for HIV has come under fierce criticism for drawing funds away from other pressing health care needs. This is not an either/or situation, nor is this argument supported by evidence [42]. An effective response to this pandemic must move beyond squabbles about AIDS versus TB, AIDS versus malaria, or AIDS versus clean safe drinking water or food security.

If the global arms trade and defence budgets were to disappear overnight and be channelled into health care for us all, these squabbles would vanish into the wind. Let's face up to the real challenges of how the wealth of our world is spent. We need more money for health care *and *more money for HIV, not less. Recent events in the world's financial markets and the responses of the rich countries provide a stark example of how governments are willing to invest huge financial resources in systems when faced with the threat of global instability. We submit that the health of the global poor, including people with HIV, constitutes an equally compelling reason to act decisively and invest in health and prosperity. Governments must request Global Fund grants to address the massive health worker shortages that are undermining all our efforts. This includes funding for recurrent costs like salaries; for efforts to reduce stigmatizing behavior by health workers; money for treatment literacy; and money for health technology currently only available in the rich nations. Universal access must mean universal health standards applicable to all, not just the fortunate few.

There is a clear need to place the expansion of health and education services at the centre of national economic planning if we are to see countries truly able to respond to HIV and AIDS. This cannot be achieved unless governments are allowed to invest in health without the restrictions imposed by international financial organizations, notably the International Monetary Fund [43,44].

Supporting the involvement of people living with HIV in health care requires drastic steps to eliminate HIV-related violence and discrimination. We have to stop violence against women [45]. We have to stop institutional violence against sex workers, drug users, men who have sex with men and transgender people. Human rights institutions, governments and justice systems need to recognize and act against the endemic violence that people with HIV are subject to day after day.

## Conclusion

People with HIV have done so much already. Through personal journeys of pain, grief, and the realisations of mortality, people with HIV have a rare gift to share with this world. In this article we have provided a brief glance at the history of involvement and suggested how this can and should be expanded. We believe this is important because participation is founded on human rights principles and in the struggle of so many, and because it constitutes a proven and vital partnership in health.

## Competing interests

The authors declare that they have no competing interests.

## Authors' contributions

All authors contributed equally to the design, structure and content of the article. All authors read and approved the final manuscript.

## References

[B1] The International Conference on Primary Health Care 1978 Declaration of Alma-Ata http://www.euro.who.int/AboutWHO/Policy/20010827_1

[B2] Joint United Nations Programme on HIV/AIDS (UNAIDS) From Principle to Practice: Greater Involvement of People Living with or Affected by HIV/AIDS (GIPA) UNAIDS, Geneva 1999

[B3] United Nations General Assembly (GA) Universal Declaration of Human Rights GA Resolution 217 A (III) of 10 December 1948 http://www.un.org/Overview/rights.html

[B4] United Nations Committee on Economic, Social and Cultural Rights General Comment No. 14 (2000) The right to the highest attainable standard of health (article 12 of the International Covenant on Economic, Social and Cultural Rights) paragraph 11 http://www.unhchr.ch/tbs/doc.nsf/(symbol)/E.C.12.2000.4.En

[B5] The Advisory Committee of the People with AIDS The Denver Principles http://www.actupny.org/documents/Denver.html

[B6] Opp Cit UNAIDS 1999

[B7] Soyinka W The Man Died 1972 Harper and Row, New York

[B8] World Health Organization (WHO) Everybody's business: strengthening health systems to improve health outcomes: WHO's framework for action, 2007 1972 http://www.searo.who.int/LinkFiles/Health_Systems_EverybodyBusinessHSS.pdf

[B9] The Collaborative Fund for HIV Treatment Preparedness Fact Sheet, nd http://www.who.int/3by5/partners/en/factsheet.pdf

[B10] Gonsalves G ART Scale up Plenary Presentation, XVII International AIDS Conference, August 2008, THPL0103 http://www.aids2008.org/Pag/PSession.aspx?s=37

[B11] Farmer P Challenging Orthodoxies: the Road Ahead for Health and Human Rights Health and Human Rights 2008 12 5 19 20845827

[B12] Uebel KE Nash J Avalos A Caring for the Caregivers: Models of HIV/AIDS Care and Treatment Provision for Health Care Workers in Southern Africa Journal of Infectious Diseases 2007 12 Suppl 3 S500 S504 10.1086/521113 18181701

[B13] Shisana O High HIV/AIDS prevalence among health workers requires urgent action S Afr Med J 2007 12 2 108 109 17404669

[B14] Odetoyinbo M Personal communication 2008

[B15] Wiel M Pos Medics Unmasked Positive Nation. London 2004 114 http://www.positivenation.co.uk/issue114/features/feature1/feature1.htm

[B16] Opp Cit Positive Medics Unmasked, Positive Nation 2004

[B17] The Elders Every human has rights, 2008 Every Human Has Rights Campaign http://www.everyhumanhasrights.org/yinka-jegede-ekpe

[B18] UNESCO and EI-EFAIDS, 2007 Supporting HIV-Positive Teachers in East and Southern Africa: Technical Consultation Report. 30 November – 1 December 2006, Nairobi, Kenya. Paris, UNESCO

[B19] UNAIDS Report on the global HIV/AIDS epidemic 2008: executive summary http://www.unaids.org/en/KnowledgeCentre/HIVData/GlobalReport/2008/2008_Global_report.asp

[B20] Human Rights Watch Locked Doors: the Human Rights of People Living with HIV/AIDS in China September 2003 http://www.hrw.org/reports/2003/china0803/china0903full.pdf

[B21] UNAIDS Report of the Meeting UNAIDS Reference Group on HIV and Human Rights. Seventh meeting 12–14 February 2007 Geneva http://data.unaids.org/pub/Report/2007/20070703_rghr7_meetingreport_en.pdf

[B22] Buskens I Jaffe A Demotivating infant feeding counselling encounters in southern Africa: Do counsellors need more or different training? AIDS Care 2008 12 3 337 345 10.1080/09540120701660346 18351482

[B23] Physicians for Human Rights Epidemic of Inequality: Women's Rights and HIV/AIDS in Botswana & Swaziland 2007 http://physiciansforhumanrights.org/library/documents/reports/botswana-swaziland-report.pdf

[B24] Amnesty International "I am at the lowest end of all": Rural women living with HIV face human rights abuses in South Africa 2008 http://www.amnesty.org/en/library/info/AFR53/001/2008/en

[B25] International Community of Women Living with HIV/AIDS (ICW) Violence against HIV Positive Women Briefing 2006 http://www.icw.org/files/Violence.pdf

[B26] ICW Concerned Over Trend to Criminalise HIV Transmission http://www.icw.org/node/354

[B27] Wright E Brew B Arayawichanont A Neurologic disorders are prevalent in HIV positive outpatients in the Asia-Pacific region Neurology 2008 12 53 59 10.1212/01.wnl.0000316390.17248.65 18591505

[B28] International AIDS Society XVII International AIDS Conference Abstract Book Volume 1 and Abstract Book Volume 2 2008 http://www.aids2008-abstracts.org/aids2008_book_vol1_web.pdf and http://www.aids2008-abstracts.org/aids2008_book_vol2_web.pdf

[B29] Fraser DE Fraser S Debating Directly Observed Therapy (DOT) and ARV Adherence among the Urban Poor 2008 http://www.aids2008.org/abstract.aspx?elementId=200721083

[B30] ICW New Research On Women's Experiences of ACTS – three reports from Kenya, Tanzania and Namibia 2006 http://www.icw.org/node/224

[B31] World Health Organization Towards universal access Scaling up priority HIV/AIDS interventions in the health sector. Progress Report 2008 http://www.who.int/hiv/mediacentre/2008progressreport/en/

[B32] Welbourn A Stepping Stones: A training package in HIV/AIDS, communication and relationship skills Oxford. Strategies for Hope 1995

[B33] Stepping Stones "Transforming Male Gender Roles" Andrew Levack Global AIDS Link 2006 12 http://www.steppingstonesfeedback.org/?page_id=287

[B34] Maher J The Impact of Increased Availability of Antiretroviral Therapy on Programmes Responding to People Living with HIV and AIDS. London. Plurpol Consulting on behalf of CAFOD 2008 http://www.plurpol.org

[B35] Kaleeba N Perspective: Raising Africa's Orphans – Seeding a "garden of hope" for children across the continent. 2008 World Pulse Magazine http://worldpulse.com/wpm/pdfs/WPM_SPRING05.pdf

[B36] Luca Susan Rader Alison Duongsaa Usa Mphuka Simon Campbell Ian Human Capacity development: learning from local action and experience. Salvation Army 2002 http://www.salvationarmy.org/ihq/www_sa.nsf/766d2187c97e6bf180256cf4005d2284/fdb5578e5e1a3c9280256f0e004aed0e/$FILE/Thai_report.pdf

[B37] Maher J The Impact of Increased Availability of Antiretroviral Therapy on Programmes Responding to People Living with HIV and AIDS. London. Plurpol Consulting on behalf of CAFOD 2008 http://www.plurpol.org

[B38] UNAIDS Policy Brief: Criminalization of HIV Transmission, 2008 http://data.unaids.org/pub/BaseDocument/2008/20080731_jc1513_policy_criminalization_en.pdf

[B39] Cameron E Criminal Statutes and Criminal Prosecutions in the Epidemic: Help or Hindrance? XVII International AIDS Conference, 2008. Plenary Presentation FRPL0103

[B40] Bactrin KM Healthworkers and HIV: Changing the tide Health Rights Advocacy Forum (HERAF) Newsletter, Health Rights Advocacy Forum, Kenya 2008 12 2

[B41] Physicians for Human Rights Bold Solutions to Africa's Health Worker Shortage. HERAF 2006 7 8

[B42] International Treatment Preparedness Coalition Treatment Monitoring & Advocacy Project. Missing the Target: The HIV Response and Health Systems: Building on success to achieve health care for all 2008 http://www.aidstreatmentaccess.org/mtt6_final.pdf

[B43] ActionAid Changing IMF Policies to Get More Doctors, Nurses and Teachers Hired in Developing Countries", 2008 http://www.ifiwatchnet.org/sites/ifiwatchnet.org/files/4-pager%20--%20IMF%20and%20health.pdf

[B44] Action Aid Confronting the Contradictions: the IMF, Wage Bill Caps and the Case for Teachers 2007 http://www.actionaid.org/assets/pdf/AAConf_Contradictions_Final2.pdf

[B45] García-Moreno C Jansen H Ellsberg M Heise L Watts C Multi-country Study on Women's Health and Domestic Violence against Women: Initial results on prevalence, health outcomes and women's responses. World Health Organization, 2005 2005 http://www.who.int/gender/violence/who_multicountry_study/en/

